# Postage stamp multiple anterior capsulorhexisotomies in pediatric cataract surgery

**DOI:** 10.1186/1471-2415-5-3

**Published:** 2005-03-08

**Authors:** Jeewan S Titiyal, Rajesh Sinha, Namrata Sharma, Rasik B Vajpayee

**Affiliations:** 1 Rajendra Prasad Centre for Ophthalmic Sciences, All India Institute of Medical Sciences, New Delhi – 110029, India

**Keywords:** postage stamp capsulotomy, anterior capsulorhexis, pediatric cataract

## Abstract

**Background:**

Capsule related complications are common following pediatric cataract surgery. We report a new technique of multiple anterior capsulorhexisotomies after lens aspiration and intraocular lens (IOL) implantation.

**Methods:**

After performing automated lens aspiration, an IOL was implanted into the capsular bag. A bent 26 gauge needle was introduced through one side port and multiple small cuts were made in one half of the circumference of the anterior capsular rim by making a radial movement of the needle tip centripetally over the margin of the anterior capsular rim. The needle was again introduced through the other side port and multiple similar cuts were made in the other half thereby creating nearly 20 – 30 cuts at the margin of the anterior capsular rim.

**Results:**

The mean size of the primary capsulorhexis was 4.33 ± 0.20 mm. A uniform enlargement of the capsulorhexis could be performed in all the eyes without peripheral extension in any of the eyes. There was no damage to the posterior capsule and no scratch mark on the IOL. In one eye, the primary capsulorhexis was slightly eccentric, though it was covering the IOL optic all around. The rhexisotomies in this eye were limited to the capsular rim that was overlapping more on the IOL optic (sectoral anterior capsulorhexisotomies).

**Conclusion:**

The technique of postage stamp anterior capsulorhexisotomies is a feasible technique in pediatric cataracts.

## Background

Pediatric cataract surgery has always been a challenge for the anterior segment surgeons and short term and long term complications have been reported in the literature [1]. Automated lens aspiration is the preferred technique employed these days for pediatric cataracts and central continuous curvilinear capsulorhexis is the method of anterior capsulotomy in this procedure. Performing a continuous curvilinear capsulorhexis (CCC) is more difficult in children than in adults because the capsular bag is more elastic. It has been reported that mechanized circular capsulectomy with a vitrector is easier to perform and safer than manual CCC in very young eyes [2].

However, most prefer using Utrata capsulorhexis forceps to complete the capsulorhexis after an initial nick in the anterior capsule with a bent 26 gauge needle.

We herein, describe a new technique of postage stamp multiple capsulorhexisotomies, a modification in the primary capsulorhexis after performing lens aspiration with intraocular lens implantation in pediatric cataracts.

## Methods

We performed this technique of anterior capsulorhexisotomies in 6 eyes of 4 children; 2 patients with bilateral developmental cataract and 2 with unilateral post-traumatic cataract.

Surgery was performed in all the eyes under general anesthesia. A clear corneal 3.2 mm 3-plane tunnel was created superiorly. Two side ports were created at 10 and 2 o'clock positions using a microvitreoretinal blade (Alcon laboratories, Fort Worth, TX). Trypan blue (Vision blue, DORC, Netherlands) was injected (0.1 ml of 0.1%) into the anterior chamber under air bubble to stain the anterior capsule and was completely washed out after 15 seconds by balanced saline solution. Anterior capsulorhexis was initiated with a bent 26 gauge needle and completed by Utrata capsulorhexis forceps to create a circular, 4.0 – 4.5 mm, central capsulorhexis. The size of the capsulorhexis was measured on a television monitor according to a previously described method [3]. Hydroprocedure was performed to soften the lens matter and a complete lens aspiration was performed using Universal II (Alcon laboratories, Fort Worth, TX) phaco machine. Vacuum cleaning of the posterior capsule and the undersurface of the anterior capsular rim was performed in all the eyes. After inflating the capsular bag with 1.4% sodium hyaluronate (Healon GV; Pharmacia & Upjohn, Kalamazoo), a single piece foldable Acrysof intraocular lens (IOL) of optic size of 6.0 mm with an overall diameter of 13.0 mm (SA60AT, Alcon laboratories) was implanted in the capsular bag and the viscoelastic substance was aspirated by rock and roll technique.

**Figure 1:**
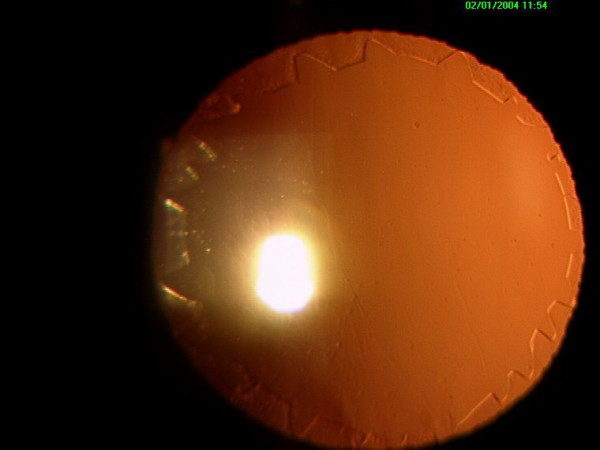
Postage stamp multiple anterior capsulorhexisotomies with in-the-bag IOL in a pediatric eye of 8 years.

Sodium hyaluronate 1.4% was injected under the anterior capsular rim. A bent 26 gauge needle was introduced through one side port and multiple small cuts were made in one half of the circumference of the anterior capsular rim by making a radial movement of the needle tip centripetally over the margin of the anterior capsular rim taking care not to put any scratch mark on the IOL. The needle was again introduced through the other side port and multiple similar cuts were made in the other half thereby creating nearly 20 – 30 cuts all around circumferentially at the margin of the anterior capsular rim (Figure 1). The viscosurgical device was aspirated by rock and roll technique and 0.1 ml of 1% vancomycin was injected intracamerally. The anterior chamber was reformed with balanced salt solution and the corneal tunnel was hydrated.

Postoperatively, patients were prescribed topical betamethasone sodium phosphate 0.1% and ciprofloxacin 0.3% QID each for 4 weeks and tropicamide 1% TID for 1 week.

## Results

The mean age of the patients was 7.87 ± 1.60 (9, 8.5, 8.5 & 5.5 years) years and all patients were males. The mean size of the primary capsulorhexis was 4.33 ± 0.20 mm. The nicks at the margin of the anterior capsular rim could be performed successfully with a bent 26 gauge needle in all the eyes. A uniform enlargement of the capsulorhexis could be performed in all the eyes without peripheral extension in any of the eyes. No eye suffered damage to the posterior capsule. There was no scratch mark of the needle on the optic of any IOL. In one eye, the primary capsulorhexis was slightly eccentric, though it was covering the IOL optic all around. The rhexisotomies in this eye were limited to the capsular rim that was overlapping more on the IOL optic (sectoral anterior capsulorhexisotomies).

## Discussion

Size of anterior capsulorhexis has always been a matter of debate for automated lens aspiration in pediatric cataracts. If the anterior capsulorhexis opening is small, there is risk of anterior capsular opacification, capsular contraction syndrome and phimosis of the anterior capsular opening, decentration of the IOL and capsular bag hyperdistension [4,5]. Despite an intact capsulorhexis, IOL decentration may still occur due to capsular contraction syndrome. If the capsulorhexis is too large, there is risk of development of adhesion between the anterior capsular rim and the posterior capsule. This can have a zipper effect on the IOL, which can result in forward popping up of the IOL and a significant change in the refractive status of the eye. The size of the anterior capsulorhexis is considered adequate when the margin of the anterior capsular rim just covers the IOL optic margin all around. However, it is very difficult to create an exactly similar size of anterior capsulorhexis in all the eyes. Intraoperative enlargement after performing lens aspiration with a previously performed smaller rhexis is possible [6-8]. But in pediatric eyes, it is very difficult to predict whether the enlarged rhexis opening will be of optimum size. In many of these situations, the size of the capsular opening enlarges more than optimum in one half or one quadrant. In an effort to prevent complications related to both smaller and larger anterior capsulorhexis, we performed multiple anterior capsulorhexisotomies like the configuration of a postage stamp, in which after creating a primary capsulorhexis of slightly smaller than optimum size, multiple nicks were made at the margin of the anterior capsular rim all around after lens aspiration and implantation of IOL. These nicks if made before lens aspiration can extend to the periphery towards the equator of the lens as the capsule is taut due to the presence of positive intralenticular pressure. However, after lens aspiration, the capsule becomes lax and hence these nicks do not extend to the periphery. If performed before IOL implantation, there is risk of damaging the posterior capsule and moreover, during IOL implantation, these nicks can extend to the periphery.

We have observed that after curvilinear capsulorhexis, capsular opacification and fibrosis is most marked at the margin of the anterior capsular rim. We performed capsulorhexisotomies to prevent phimosis of the anterior capsular opening and capsular contraction syndrome, as these small nicks act as relaxing incisions. Since there were multiple nicks all around, the direction of the force is well distributed. The vector of the force generated at the opacified margin of curvilinear capsulorhexis is directed inwards, while after performing multiple nicks in this margin (capsulorhexisotomies), the vector is directed in both inward as well as outward directions. Therefore, there is less chance of development of capsular contraction syndrome. More over, there is negligible chance of adhesion between the anterior and posterior capsules as the anterior capsular rim rests well on the IOL optic.

## Conclusion

The postage stamp multiple anterior capsulorhexisotomies is a feasible and safe technique after lens aspiration and IOL implantation in pediatric cataracts.

## Declaration of competing interest

The author(s) declare that they have no competing interests.

## Individual contribution of authors

JST designed the study and performed surgeries. RS performed the data collection and wrote the manuscript. NS followed up the patients. RBV performed the surgeries.

All authors read and approved the final manuscript.

## Pre-publication history

The pre-publication history for this paper can be accessed here:
